# Muscle metabolic and neuromuscular determinants of fatigue during cycling in different exercise intensity domains

**DOI:** 10.1152/japplphysiol.00942.2016

**Published:** 2016-12-22

**Authors:** Matthew I. Black, Andrew M. Jones, Jamie R. Blackwell, Stephen J. Bailey, Lee J. Wylie, Sinead T. J. McDonagh, Christopher Thompson, James Kelly, Paul Sumners, Katya N. Mileva, Joanna L. Bowtell, Anni Vanhatalo

**Affiliations:** ^1^College of Life and Environmental Sciences, St. Luke’s Campus, University of Exeter, Exeter, United Kingdom;; ^2^School of Sport, Exercise and Health Sciences, Loughborough University, United Kingdom; and; ^3^Sport and Exercise Science Research Centre, School of Applied Sciences, London South Bank University, London, United Kingdom

**Keywords:** critical power, gas exchange threshold, neuromuscular fatigue, muscle metabolism, cycling exercise

## Abstract

Lactate or gas exchange threshold (GET) and critical power (CP) are closely associated with human exercise performance. We tested the hypothesis that the limit of tolerance (T_lim_) during cycle exercise performed within the exercise intensity domains demarcated by GET and CP is linked to discrete muscle metabolic and neuromuscular responses. Eleven men performed a ramp incremental exercise test, 4–5 severe-intensity (SEV; >CP) constant-work-rate (CWR) tests until T_lim_, a heavy-intensity (HVY; <CP but >GET) CWR test until T_lim_, and a moderate-intensity (MOD; <GET) CWR test until T_lim_. Muscle biopsies revealed that a similar (*P* > 0.05) muscle metabolic milieu (i.e., low pH and [PCr] and high [lactate]) was attained at T_lim_ (approximately 2–14 min) for all SEV exercise bouts. The muscle metabolic perturbation was greater at T_lim_ following SEV compared with HVY, and also following SEV and HVY compared with MOD (all *P* < 0.05). The normalized M-wave amplitude for the vastus lateralis (VL) muscle decreased to a similar extent following SEV (−38 ± 15%), HVY (−68 ± 24%), and MOD (−53 ± 29%), (*P* > 0.05). Neural drive to the VL increased during SEV (4 ± 4%; *P* < 0.05) but did not change during HVY or MOD (*P* > 0.05). During SEV and HVY, but not MOD, the rates of change in M-wave amplitude and neural drive were correlated with changes in muscle metabolic ([PCr], [lactate]) and blood ionic/acid-base status ([lactate], [K^+^]) (*P* < 0.05). The results of this study indicate that the metabolic and neuromuscular determinants of fatigue development differ according to the intensity domain in which the exercise is performed.

**NEW & NOTEWORTHY** The gas exchange threshold and the critical power demarcate discrete exercise intensity domains. For the first time, we show that the limit of tolerance during whole-body exercise within these domains is characterized by distinct metabolic and neuromuscular responses. Fatigue development during exercise greater than critical power is associated with the attainment of consistent “limiting” values of muscle metabolites, whereas substrate availability and limitations to muscle activation may constrain performance at lower intensities.

## Abstract (precis)

The gas exchange threshold and the critical power demarcate discrete exercise intensity domains. For the first time, we show that the limit of tolerance during whole body exercise within these domains is characterized by distinct metabolic and neuromuscular responses. Fatigue development during exercise greater than critical power is associated with the attainment of consistent “limiting” values of muscle metabolites, whereas substrate availability and limitations to muscle activation may constrain performance at lower intensities.

intense and/or prolonged excitation of muscle leads to a reversible decline in its force-generating capacity and rate of contraction, commonly known as fatigue (21–23, 27, 55). This temporary reduction in muscle performance may be attributed to central factors that limit the neural drive for muscle contraction, and to peripheral factors that occur at or distal to the neuromuscular junction and that often involve metabolic and ionic perturbations that reduce the muscle’s ability to respond to neural stimulation (2, 3, 25, 30, 41).

The extent of the muscle metabolic and ionic, and blood acid-base and respiratory perturbations experienced during exercise is dependent on exercise intensity, which can be categorized into three distinct domains demarcated by physiological thresholds (32, 72). The upper limit of the moderate exercise-intensity domain is indicated by the lactate threshold [LT, which is often estimated using the gas exchange threshold (GET)], and the boundary between the heavy and severe exercise-intensity domains is given by the critical power (CP). Using ^31^P-magnetic resonance spectroscopy, it has been demonstrated that severe-intensity, single-leg knee-extension exercise is associated with a progressive loss of muscle homeostasis with time {i.e., progressive reductions in muscle phosphocreatine concentration ([PCr]) and pH, and an increase in inorganic phosphate concentration ([P_i_])} (9, 31, 33, 69). In contrast, heavy- and moderate-intensity small-muscle-mass exercise is associated with much more limited muscle metabolic perturbation with new steady-state values of [PCr], pH, and [P_i_] being achieved within a few minutes of initiation of exercise (33, 48, 67). These intensity-related differences in muscle metabolic, as well as related blood acid-base and respiratory gas exchange responses to exercise (33, 51, 68, 73), likely underpin the close relationships reported between these threshold phenomena (LT/GET, and CP) and human exercise performance (8).

The role of exercise intensity in defining the extent and dynamics of muscle metabolic perturbation implies that exercise intensity may also influence the nature of neuromuscular fatigue development (3, 22, 24, 39, 41, 52, 53). The peripheral component to fatigue, as estimated noninvasively using surface electromyography (EMG), electrical muscle stimulation, transcranial magnetic stimulation, or a combination of these appears to be especially important during high-intensity exercise (45, 64, 66), whereas central fatigue may be more prominent during prolonged, low-intensity exercise (38, 45, 57, 61, 66). The intensity-dependent interaction between peripheral and central components of fatigue is thought to be modulated by changes in afferent feedback arising from the muscle metabolic milieu. Consistent with this, the critical torque (CT; analogous with CP) for small-muscle-mass exercise has been shown to represent a threshold in the development of neuromuscular fatigue (10), such that severe-intensity knee-extensor contractions (>CT) were associated with elevated motor unit recruitment and a disproportionate increase in the rate of neuromuscular fatigue development relative to heavy-intensity contractions (<CT).

It is presently unclear whether the determinants of neuromuscular fatigue development during whole body exercise such as cycling differ according to the intensity domain in which the exercise is performed. Previous studies have assessed neuromuscular fatigue before and after self-paced maximal time-trial cycle exercise (66) and during constant work rate (CWR) cycling performed ostensibly within the severe-intensity domain (65). These studies suggested that, in contrast to knee extension exercise (10), the level of peripheral fatigue at exhaustion for cycling may also be intensity-dependent above CP (65). Compared with small-muscle-mass exercise, whole body exercise is associated with greater rates of pulmonary ventilation and gas exchange (58, 74), differences in cardiac output and muscle perfusion (12, 46, 58), and greater activity of type III/IV muscle afferents that may modulate central drive (52, 53). It is possible that these factors affect the relationship between muscle metabolic changes and neuromuscular fatigue development during exercise.

To date, the physiological and neuromuscular responses to whole body exercise and their possible interrelationship has not been assessed within distinct exercise intensity domains. The purpose of this study therefore was to evaluate possible differences in the muscle metabolic and systemic responses to different, well-defined intensities of exercise, with the aim of elucidating whether the exercise intensity domain influences the determinants of neuromuscular fatigue. Based on earlier studies investigating small-muscle-mass exercise (33, 69), we tested the hypotheses that *1*) a consistent muscle metabolic milieu ([ATP], [PCr], [lactate], pH) and neuromuscular responses (muscle excitability and neural drive) will be attained at the limit of tolerance (T_lim_) during severe-intensity exercise (>CP); *2*) severe-intensity exercise will be associated with greater muscle metabolic perturbation compared with heavy- and moderate-intensity exercise; and *3*) the rate of neuromuscular fatigue development will be greater during severe- compared with heavy- and moderate-intensity exercise due to greater muscle metabolic and ionic perturbations.

## METHODS

### Ethical Approval

The protocols were approved by the Research Ethics Committee of Sport and Health Sciences (University of Exeter) and conducted in accordance with the code of the ethical principles of the World Medical Association (Declaration of Helsinki). Subjects gave written informed consent to participate after the experimental procedures, associated risks, and potential benefits of participation had been explained.

### Subjects

Eleven healthy recreationally active men (age, 21.8 ± 1.9 yr; height, 1.79 ± 0.05 m; body mass, 78.2 ± 8.1 kg; means ± SD) volunteered to participate in this study, 8 of whom volunteered to provide muscle tissue samples. One subject who volunteered for the biopsy procedure withdrew from the study after completing only the severe-intensity exercise trials. That subject’s data were excluded from statistical difference tests, but included in the correlational analysis. All subjects were in good health and had no known history of neurological or motor disorder. Subjects were instructed to report to all testing sessions in a rested and fully hydrated state ≥3 h postprandial, and to avoid strenuous exercise and refrain from caffeine and alcohol in the 24 h before testing. Each subject started each experimental trial at the same time of day (±2 h). All trials were performed on the same electronically braked cycle ergometer (Lode; Excalibur, Groningen, The Netherlands).

### Experimental Design

**Fig. 1.**
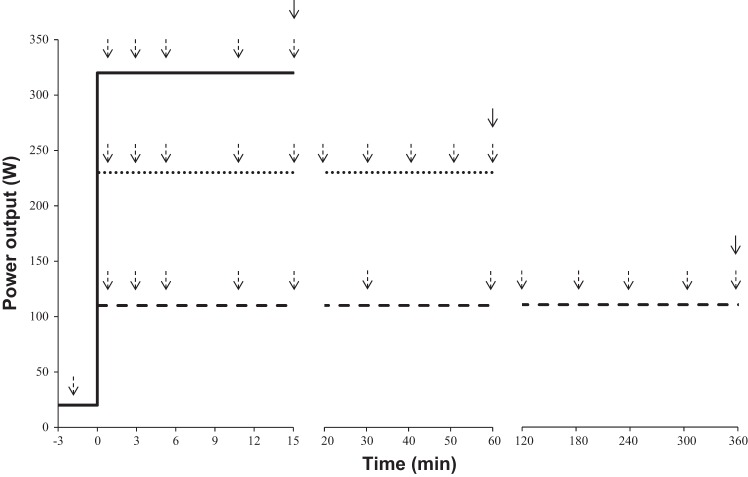
Schematic of the exercise protocol. Group mean work rates are shown for the severe- (solid line), heavy- (dotted line), and moderate- (dashed line) intensity trials. All trials were started with a 3-min warm-up phase at 20 W, followed by an immediate step increase to the required work rate. Subjects were encouraged to continue exercising as long as possible. Dashed arrows indicate collection of venous blood and femoral nerve stimulation. Solid arrows indicate collection of muscle tissue. For clarity, the resting muscle sample obtained before the first trial is not shown.

Each subject visited the laboratory on approximately seven occasions over a 6-wk period with each visit separated by a minimum of 24 h. A minimum recovery of 7 days was provided following the heavy- and moderate-intensity exercise tests. After the completion of a ramp incremental test (*visit 1*), subjects performed four to five CWR severe-intensity exercise tests to define the power-duration relationship, a heavy-intensity CWR test, and a moderate-intensity CWR test, completed in a randomized order (Fig. 1), except that the severe-intensity tests always preceded the heavy-intensity test. Pulmonary gas exchange was measured continuously during all tests, except the moderate-intensity test, during which it was measured periodically for 10-min intervals, with the midpoint of collection coinciding with blood sample collection and femoral nerve stimulation (see below). We encouraged the subjects to continue exercising during the moderate-intensity test to enable 10 min of gas exchange data to be collected immediately before exercise cessation. EMG data were obtained continuously from the vastus lateralis (VL) and vastus medialis (VM) muscles throughout the exercise period with stimulation of the femoral nerve delivered at regular intervals (Fig. 1) to quantify the neuromuscular changes occurring during the exercise protocols. Venous blood samples were obtained before and during exercise for the moderate-, heavy-, and for three of the severe-intensity exercise tests. In addition, muscle tissue was obtained at rest and immediately following the moderate-, heavy-, and three of the severe-intensity exercise tests (Fig. 1). The severe-intensity tests were performed at three to five different work rates spanning 60%Δ to V̇o_2peak_ (where Δ refers to the work rate difference between GET and V̇o_2peak_). Three of these severe-intensity tests (including short 85 ± 5%Δ, intermediate 75 ± 5%Δ, and long 65 ± 5%Δ) were grouped and compared for differences in muscle, neuromuscular, and blood responses within the severe-intensity domain.

### Incremental Test

During the first laboratory visit, subjects completed a ramp incremental test for determination of the V̇o_2peak_ and GET. The ergometer seat height and handlebars were adjusted for comfort, and the same settings were replicated for each subsequent test. Initially, subjects completed 3 min of baseline cycling at 20 W, after which the work rate was increased by 30 W/min until volitional exhaustion. Subjects cycled at a constant self-selected pedal rate [80 revolutions per minute (rpm), *n* = 9, 90 rpm, *n* = 2), which was recorded and reproduced in subsequent tests. The test was terminated when the pedal rate fell more than 10 rpm below the preferred value for more than 5 s despite strong verbal encouragement. Breath-by-breath pulmonary gas exchange data were collected continuously throughout the test and recorded as a 10-s moving average for data analysis. V̇o_2peak_ was determined as the highest mean V̇o_2_ during any 30-s period, and GET was determined as previously described (5, 68).

### CWR Tests

All CWR tests started with 3 min of cycling at 20 W, followed by a step increase to the required work rate. Subjects were instructed to remain seated and to maintain their preferred pedal rate for as long as possible. Strong verbal encouragement was provided, but subjects were not informed of either the work rate or the elapsed time. Tests were terminated when pedal rate fell more than 10 rpm below the preferred value for more than 5 s. T_lim_ was recorded to the nearest second.

The parameters of the power-duration relationship (CP and W′) were estimated by completion of four to five severe-intensity exercise tests (4 trials, *n* = 9; 5 trials *n* = 2) at different work rates (~60%Δ, 70%Δ, 80%Δ, and 100% V̇o_2peak_) resulting in T_lim_ ranging between ~2 and 14 min. If the standard errors associated with the CP and W′ exceeded 5 and 10%, respectively, after four exercise tests had been completed, a fifth test was performed. Any tests in which the end-exercise V̇o_2_ was <95% of the individual’s ramp test determined V̇o_2peak_ were excluded from the modeling of the power-duration relationship.

CP and W′ (the amount of work performed above the CP) parameters were estimated using three models: the hyperbolic P-T_lim_ model (Eq. 1); the linear work-time (W-T_lim_) model, in which the total work performed (W) is plotted against time (Eq. 2); and the linear inverse-of-time (1/T_lim_) model, in which power output is plotted against the inverse of time (Eq. 3):

(1) $$T_{lim}=\mathrm{W\prime}/\left(P–CP\right)$$

(2) $$W=CP\;\cdot \;T_{lim}+W\prime$$

(3) $$P=\mathrm{W\prime}\left(1/T_{lim}\right)+CP$$

The standard errors of the estimate associated with CP and W′ were expressed as coefficients of variation (CV%; that is, relative to the parameter estimate). For each subject, the “best fit” model associated with the lowest CV% for CP and W′ was used for further analyses (7).

The work rate for the heavy-intensity CWR trial was equal to the lower bound of the 95% confidence limit in the CP parameter (33). The moderate-intensity CWR trial was performed at a work rate corresponding to 90% of GET. Subjects were permitted to ingest water ad libitum during the heavy- and moderate-intensity tests.

### Pulmonary Gas Exchange

Breath-by-breath pulmonary gas exchange and ventilation were measured continuously during all exercise tests, except the moderate-intensity test, when it was measured at discrete time points. Subjects wore a nose clip and breathed through a mouthpiece and impeller turbine assembly (Jaeger Triple V; Jaeger, Hoechberg, Germany). The inspired and expired gas volume and concentration signals were continuously sampled at 100 Hz (Oxycon Pro; Jaeger) via a capillary line connected to the mouthpiece. The gas analyzers were calibrated before each trial with gases of known concentration, and the turbine volume transducer was calibrated using a 3-liter syringe (Hans Rudolph, Kansas City, MO). The volume and concentration signals were time aligned by accounting for the delay in capillary gas transit and the analyzer rise time relative to the volume signal.

### Blood Analyses

Venous blood samples were drawn into 5-ml heparinized syringes (Terumo, Leuven, Belgium) from a cannula (Insyte-W; Becton Dickinson, Madrid, Spain) inserted into the subject’s antecubital vein. Blood was analyzed for [lactate] within ~5 min of collection (YSI 2300; Yellow Springs Instruments, Yellow Springs, OH). The remaining whole blood was then centrifuged at 4,000 rpm for 7 min (Hettich EBA 20; Germany) before plasma was extracted and analyzed for [K^+^] (9180 Electrolyte Analyzer; F. Hoffman-La Roche, Basel, Switzerland).

### Neuromuscular Function

EMG was used to continuously record VL and VM activity during exercise using active bipolar bar electrodes with single differential configuration (DE2.1; DelSys, Boston, MA), positioned over the muscle belly [surface EMG for noninvasive assessment of muscles (SENIAM) guidelines]. The ground electrode was positioned on the patella. Double-sided adhesive interfaces and hypoallergenic medical tape were used to keep the EMG sensors in place and to reduce skin impedance. The leads connected to the electrodes were secured using hypoallergenic medical tape to minimize artifacts due to movement of the leads. The skin area underneath each electrode was shaved, abraded, and cleaned with alcohol swabs before electrode placement to minimize skin impedance. The EMG signal was considered of good quality when the average rectified EMG baseline level for each muscle was below 2 μV (18). The EMG signals were preamplified (1,000×), band-pass filtered (20–450 Hz, Bagnioli-8; DelSys), and digitized at a sampling rate of 2,000 Hz and resolution of 16 bits using a Power 1401 mk-II analog-to-digital converter and Spike 2 data collection software run by custom-written sampling configuration (CED; Cambridge Electronic Design, UK).

The location of the optimal site for transcutaneous femoral nerve stimulation was determined while subjects were positioned on the cycle ergometer. Using an adhesive cathode (Boots UK, Nottingham, England) placed ~2 cm medial of the femoral pulse, and an adhesive anode (Boots UK) placed at the anterior aspect of the iliac crest, single electrical pulses generated by a constant current stimulator (DS7 A; Digitimer, UK) were delivered. The cathode was systematically moved vertically and horizontally, and the amplitude of the compound muscle action potential (i.e., M-wave) was monitored to identify the optimal position of the cathode for attaining maximal peak-to-peak M-wave amplitude during the cycling trials.

Following attachment of EMG and stimulation electrodes, the crank angle at which stimulation was to be delivered during the trials was determined for each subject. Subjects were positioned on the cycle ergometer and cycled at a moderate work rate (20 W below GET) for 1 min. EMG activity obtained during this period was rectified and averaged for 20 complete crank revolutions. The duration of each revolution was determined by a custom-made magnetic switch that generated an event marker signal on each occasion that the crank passed top dead center (i.e., 0°). For each subject, the crank angle at which the rectified VL EMG activity was maximal was determined, and as performed by Sidhu et al. (56), stimulations were delivered at the identified crank angle for all subsequent trials for that participant (65 ± 5° relative to the top dead center). A custom-written sequencer script triggered three stimulations, with at least 1 and up to 10 pedal revolutions between stimuli. The intervals were randomly determined using a random number generator incorporated within the sequencer script. This was designed to prevent participants from anticipating the stimulus delivery, which may have affected the evoked response.

A standard M-wave recruitment curve protocol was completed during each laboratory visit. Subjects cycled at 20 W below GET throughout the recruitment curve protocol. A single-pulse electrical stimulation (200 µs) was delivered at the individually identified crank angle as described above. The current was increased in 20-mA increments until the M-wave amplitude reached a plateau at the maximal M-wave amplitude (M_max_). A pulse of 130% M_max_ current was applied during the exercise tests (mean stimulation intensity, 350 ± 50 mA).

EMG signals from the VL and VM were processed using a custom-written script to measure peak-to-peak M-wave amplitude and M-wave area. The root mean square (RMS) of the EMG signal (an index of the power of the signal) was calculated as the mean over a 25-ms prestimulation period at each stimulation time point. The EMG RMS amplitudes and the M-wave parameters were normalized to the corresponding values attained after 1 min of exercise during each trial to evaluate temporal changes in the voluntary muscle activation level (i.e., EMG RMS amplitude) and the peripheral neuromuscular excitability (i.e., M-wave amplitude and area). In addition, the voluntary EMG RMS amplitude was normalized to the M-wave amplitude recorded at that time point to assess changes in neural drive (RMS/M; 42). The rates of change in M-wave and EMG parameters from baseline cycling to T_lim_ were calculated for each exercise to quantify the rate of neuromuscular fatigue development in each intensity domain.

### Muscle Biopsy

The biopsy site was prepared on the alternate thigh to the EMG and peripheral nerve stimulation setup. Local anesthesia was applied (2–3 ml of 20 mg/ml lidocaine) and an incision was made in the medial region of the VL. Muscle samples were obtained using needle biopsy with suction (6). Resting muscle samples were obtained before any exercise on the first laboratory visit and postexercise biopsies were taken within ~10 s of cessation of each exercise test with the subject supported on the ergometer. Muscle tissue was rapidly frozen in liquid nitrogen.

### Muscle Tissue Analysis

Frozen muscle samples from each biopsy were weighed before and after freeze-drying to determine water content. After freeze-drying, the muscle samples were dissected free from blood, fat, and connective tissue. Before muscle metabolite analysis, 200 µl of 3 M perchloric acid was added to ~2.5 mg of dry weight (dw) muscle. The solution was then centrifuged and placed on ice for 30 min. It was subsequently neutralized to pH 7.0 with 255 µl of cooled KHCO_3_ and centrifuged (10,000 *g*). The supernatant was analyzed for PCr, ATP, and lactate by fluorometric assays (35). An aliquot containing 1–2 mg of dw muscle was extracted in 1 M HCl and hydrolyzed at 100°C for 3 h before glycogen content was determined using the hexokinase method (35). Muscle pH was measured using a glass electrode following the homogenization of 1–2 mg of dw of muscle in a nonbuffering solution containing (in mM) 145 KCl, 10 NaCl, and 5 iodoacetic acid.

### Statistical Analyses

One-way ANOVA with repeated measures was used to assess differences between severe-intensity exercise tests in V̇o_2peak_, muscle [ATP], [PCr], [pH], [lactate], and [glycogen], M-wave amplitude, M-wave area, voluntary EMG amplitude and RMS/M, and blood and plasma variables at T_lim_. The data from the severe-intensity tests were subsequently averaged for each subject for comparison with the heavy- and moderate-intensity tests. Differences in V̇o_2peak_, muscle [ATP], [PCr], [pH], [lactate], and [glycogen] between the severe-, heavy-, and moderate-intensity tests were assessed using a one-way ANOVA. Two-way repeated-measures ANOVA (condition × time) was used to analyze differences in M-wave amplitude and area, and voluntary EMG amplitude for VL and VM muscles, and blood and plasma variables at common time points (baseline, 1 min, 3 min, and T_lim_) among the severe-, heavy-, and moderate-intensity tests. Significant interaction and main effects were followed up with a Bonferroni post hoc test. Relationships between the rates of change of metabolic and neuromuscular variables were assessed using Pearson’s product-moment correlation coefficients. Statistical significance was set at *P* < 0.05 and data are presented as means ± SD.

## RESULTS

The V̇o_2peak_ measured in the ramp incremental test was 4.32 ± 0.46 l/min (56 ± 8 ml·kg^−1^·min^−1^) and the peak work rate was 385 ± 50 W. GET occurred at 2.33 ± 0.34 l/min and 137 ± 24 W.

### Physiological Responses Within the Severe-Intensity Domain

T_lim_ values in the severe-intensity CWR exercise tests ranged from 2.2 to 13.9 min. There were no differences between the three models (Eqs. 1–3) in the CP or W′ estimates (*P* > 0.05; Table 1). The CP from the best-fit model corresponded to 64 ± 7% of ramp test peak work rate and 45 ± 11%Δ.

**Table 1.** Parameter estimates derived from Eqs. 1–3 and the “optimized fit” model

	*R*^2^	CP, W	SEE, W	CV%	W′ kJ	SEE, kJ	CV%
W-T_lim_ model	0.993–1.000	253 ± 54	6 ± 3	2.6 ± 1.4	22.5 ± 5.3	2.3 ± 1.0	11.0 ± 6.2
1/T_lim_ model	0.939–0.999	252 ± 52	7 ± 4	3.0 ± 2.3	20.7 ± 5.2	1.9 ± 1.1	9.5 ± 5.6
P-T_lim_ model	0.919–1.000	248 ± 52	5 ± 3	2.2 ± 1.4	22.4 ± 3.8	2.5 ± 1.8	11.3 ± 9.4
Optimized fit model	0.944–1.000	250 ± 53	5 ± 2	2.0 ± 1.2	22.5 ± 6.1	1.8 ± 0.8	8.3 ± 4.5

CP, critical power; CV%, coefficient of variation; SEE, standard error of estimate; W′ finite work capacity above the CP.

**Fig. 2.**
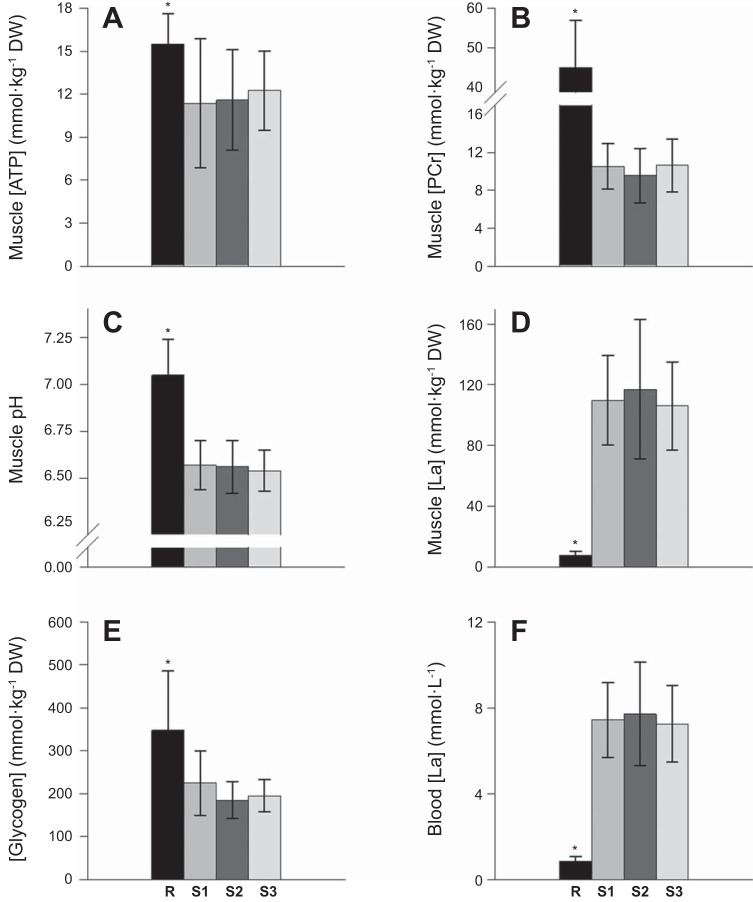
Muscle metabolic responses ([ATP] (*A*), phosphocreatine ([PCr]) (*B*), pH (*C*), [lactate] (*D*), [glycogen] (*E*), and blood [lactate] (*F*) at the limit of tolerance (T_lim_) were not different following exhaustive exercise at three different severe-intensity work rates. R, rest; S1, short trials at ~85%Δ (T_lim_ = 224 ± 41 s); S2, intermediate trials at ~75%Δ (T_lim_ = 333 ± 131 s); and S3, long trials at ~65%Δ (T_lim_ = 475 ± 145 s). *Different from S1, S2, and S3 (*P* < 0.05).

V̇o_2peak_ values during the shorter (~85%Δ, 4.43 ± 0.50 l/min), intermediate (~75%Δ, 4.49 ± 0.47 l/min), and longer (~65%Δ, 4.41 ± 0.47 l/min) severe-intensity tests were not different from V̇o_2peak_ achieved during the ramp incremental test (all *P* > 0.05). Moreover, no significant differences were observed at T_lim_ among the three severe-intensity tests for any of the muscle tissue variables or for blood [lactate] (all *P* > 0.05; Fig. 2). There were also no differences in plasma [K^+^] at T_lim_ among the shorter (5.6 ± 0.6 mM), intermediate (5.8 ± 1.1 mM), and longer (5.7 ± 0.6 mM) severe-intensity tests (*P* > 0.05).

### Physiological Responses During Severe-, Heavy-, and Moderate-Intensity Exercise

**Fig. 3.**
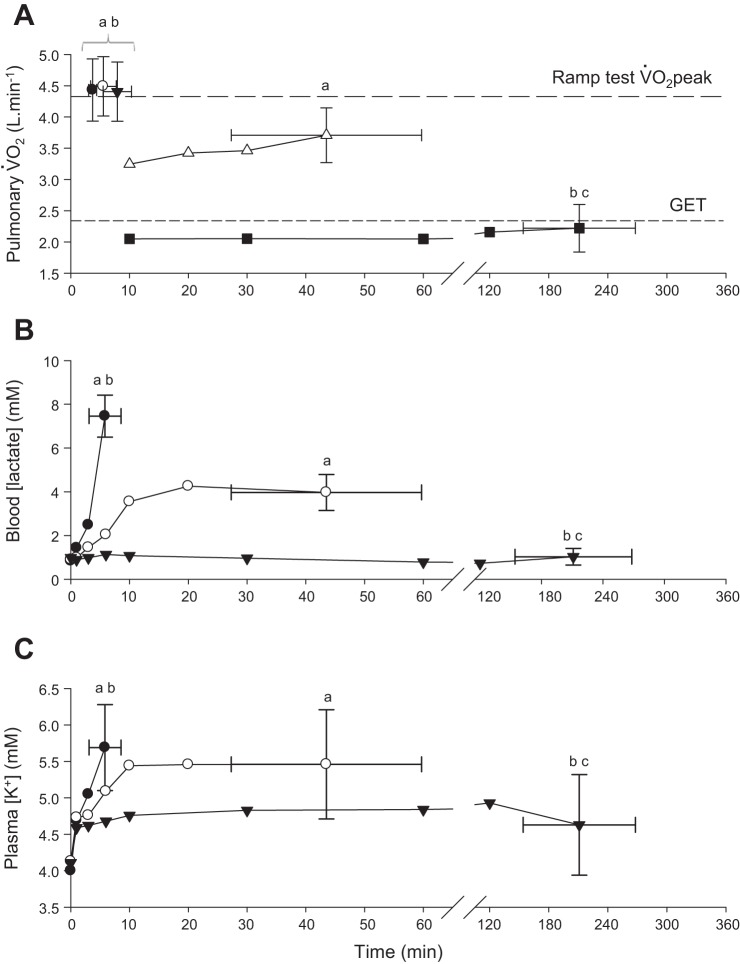
Pulmonary V̇o_2_ (*A*) during severe (solid circle, open circle, solid triangle)-, heavy (open triangles)-, and moderate (solid squares)-intensity exercise. End-exercise VO_2_ values in the three severe-intensity trials were not different from the ramp test VO_2_peak. Blood [lactate], (*B*), and plasma [K^+^] (*C*) responses to severe- (solid circles), heavy- (open circles), and moderate- (solid triangles) intensity exercise. To aid clarity error bars have been omitted from all but the final data point. ^a^Different from moderate-intensity *P* < 0.05; ^b^different from heavy-intensity *P* < 0.05; ^c^different from severe-intensity (*P* < 0.05).

Pulmonary V̇o_2_, blood [lactate], and plasma [K^+^] during moderate-, heavy-, and severe-intensity exercise are illustrated in Fig. 3. The T_lim_ for heavy-intensity exercise (231 ± 56 W) was 43.5 ± 16.2 min (range, 20.5 to 67.4 min), and V̇o_2_ at T_lim_ (3.78 ± 0.53 l/min; 87 ± 4% of V̇o_2peak_) was lower than the ramp test V̇o_2peak_ (*P* < 0.05). T_lim_ for the moderate-intensity exercise (113 ± 19 W) was 211.1 ± 57.0 min (range, 180 to 360 min) and the V̇o_2_ at T_lim_ (2.22 ± 0.38 l/min, 52 ± 8% of V̇o_2peak_) was not different from GET (*P* > 0.05). In 9 of 11 subjects V̇o_2_ remained below GET throughout the moderate-intensity exercise bout.

During severe-intensity exercise, blood [lactate] increased rapidly until T_lim_ and was significantly greater than baseline values after 3 min (*P* < 0.05). During heavy-intensity exercise, the rate of blood [lactate] increase was slower than during severe-intensity exercise such that blood [lactate] did not differ from baseline until after 10 min (*P* < 0.05), and no further increase was observed between 10 min and T_lim_ (*P* > 0.05) (Fig. 3*B*). Plasma [K^+^] was elevated above baseline at all measurement time points during heavy- and severe-intensity exercise (all *P* < 0.05). The [K^+^] continued to rise throughout severe-intensity exercise, although it stabilized during heavy-intensity exercise beyond 6 min (Fig. 3*C*). During moderate-intensity exercise, blood [lactate] did not change from baseline (*P* > 0.05), although plasma [K^+^] was elevated above resting baseline at 1 min (*P* < 0.05), with no further increase thereafter (all time points *P* > 0.05).

**Fig. 4.**
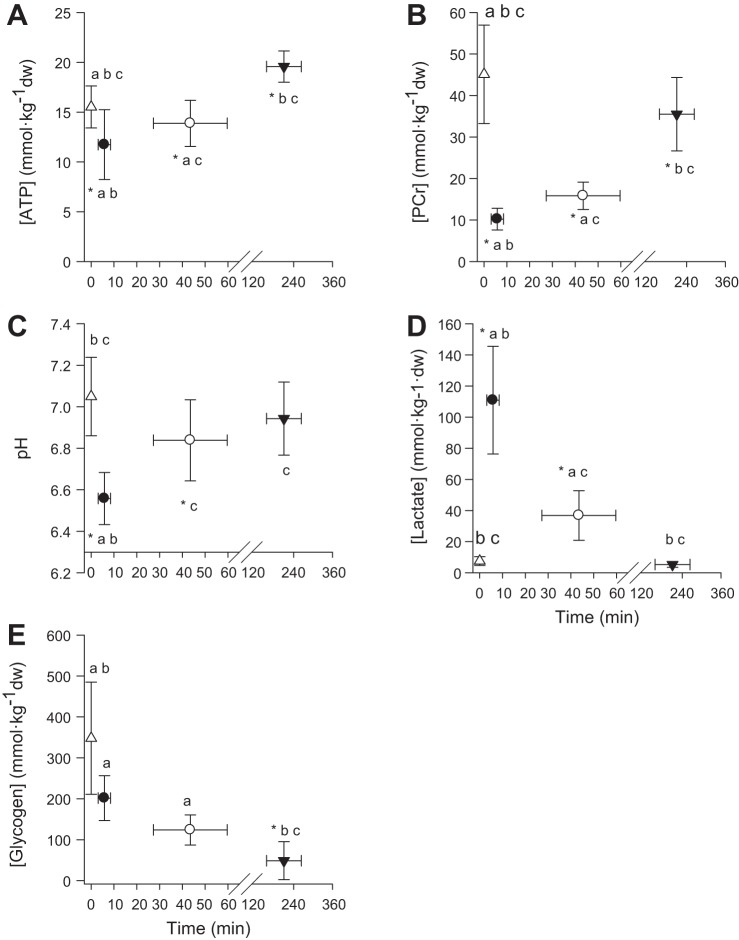
Muscle [ATP] (*A*), [PCr] (*B*), [pH] (*C*), [lactate] (*D*), and [glycogen] (*E*) at rest (open triangles), and following severe- (solid circles), heavy- (open circles), and moderate-intensity exercise (solid triangles). *Different from rest *P* < 0.05. ^a^Different from moderate-intensity *P* < 0.05; ^b^different from heavy-intensity *P* < 0.05; ^c^different from severe-intensity *P* < 0.05.

Muscle metabolic variables at rest and at T_lim_ following moderate-, heavy-, and severe-intensity exercise are illustrated in Fig. 4. For severe- and heavy-intensity exercise, muscle [ATP], [PCr], and pH were lower, and muscle [lactate] was greater at T_lim_ relative to rest (all *P* < 0.05). There was no significant muscle [glycogen] depletion during severe-intensity exercise relative to rest (*P* > 0.05), but there was a tendency for glycogen depletion during heavy-intensity exercise (*P* = 0.06). In contrast, for moderate-intensity exercise, muscle [PCr] at T_lim_ was greater than for severe- and heavy-intensity exercise (all *P* < 0.05), and muscle [glycogen] was both lower than at rest and lower than at T_lim_ for heavy- and severe-intensity exercise (all *P* < 0.05). Muscle [pH] and [lactate] did not change significantly from rest during moderate-intensity exercise (*P* > 0.05).

### Neuromuscular Responses During Severe-, Heavy-, and Moderate-Intensity Exercise

The coefficients of variation (CV%) between trials during unloaded cycling were 25% (VL) and 35% (VM) for peak-to-peak M-wave amplitude, and 32% (VL) and 32% (VM) for M-wave total area. CV% between stimulations during unloaded cycling was 11% (VL) and 9% (VM) for peak-to-peak M-wave amplitude, and 10% (VL) and 9% (VM) for M-wave total area. Mean M_max_ amplitudes measured during cycling at 20 W below GET (VL, 2.77 ± 1.43; VM, 0.99 ± 1.18 mV) were not different between visits (all *P* > 0.05). No significant differences were observed between trials in the neural drive to VL and VM during cycling at 20 W below GET.

#### Neuromuscular excitability: M-wave amplitude and M-wave area.

**Fig. 5.**
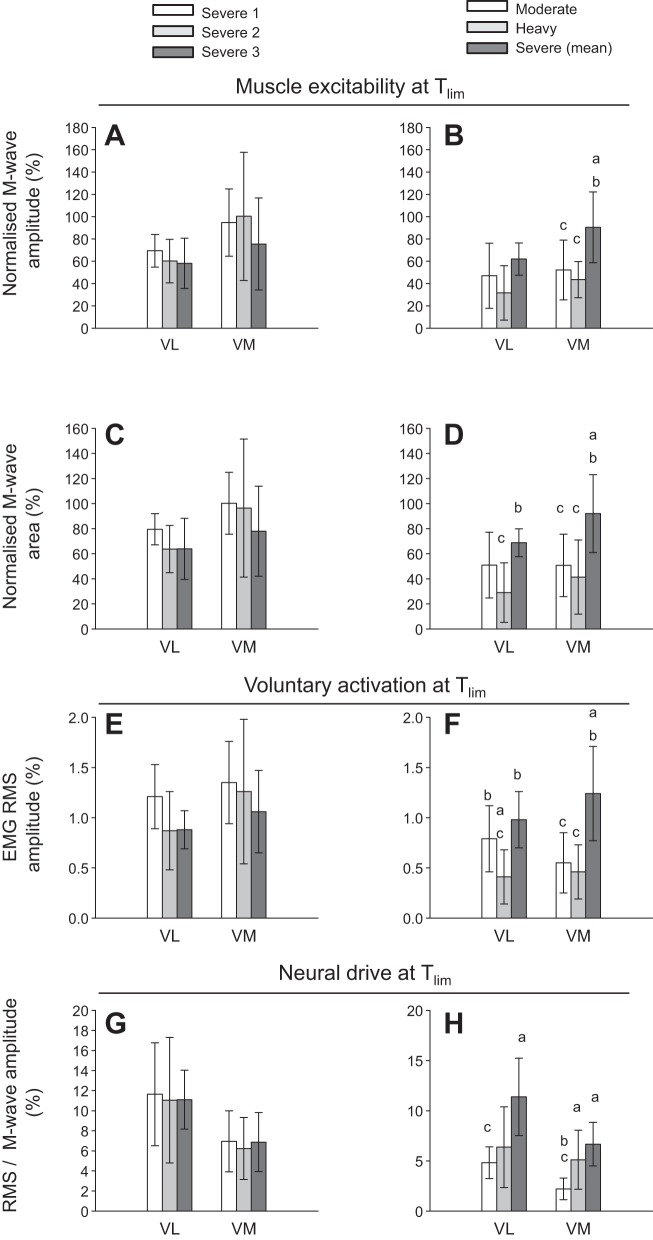
Compound muscle action potential (M-wave) amplitude and M-wave area (normalized to maximum M-wave during baseline pedalling) (group means ± SD) indicating peripheral neuromuscular excitability (*A–D*); voluntary electromyographic root mean square (EMG RMS) amplitude (normalized to M-wave amplitude at 1 min of exercise) indicating muscle activation level (*E* and *F*); and RMS/M-wave (normalized to corresponding M-wave amplitude at each measurement time point) indicating central fatigue (*G* and *H*) at the limit of tolerance (T_lim_) for moderate-, heavy-, and severe-intensity exercise (*B*, *D*, *F*, and *H*) and for three work rates (severe 1 ~85%Δ, severe 2 ~75%Δ, and severe 3 ~65%Δ) within the severe-intensity domain (*A*, *C*, *E*, and *G*). There were no significant differences among the severe-intensity work rates in muscle excitability (*A* and *C*) or indices of central fatigue (*E* and *G*). VL, vastus lateralis muscle; VM, vastus medialis muscle. ^a^Different from moderate-intensity *P* < 0.05; ^b^different from heavy-intensity *P* < 0.05; ^c^different from severe-intensity *P* < 0.05.

**Fig. 6.**
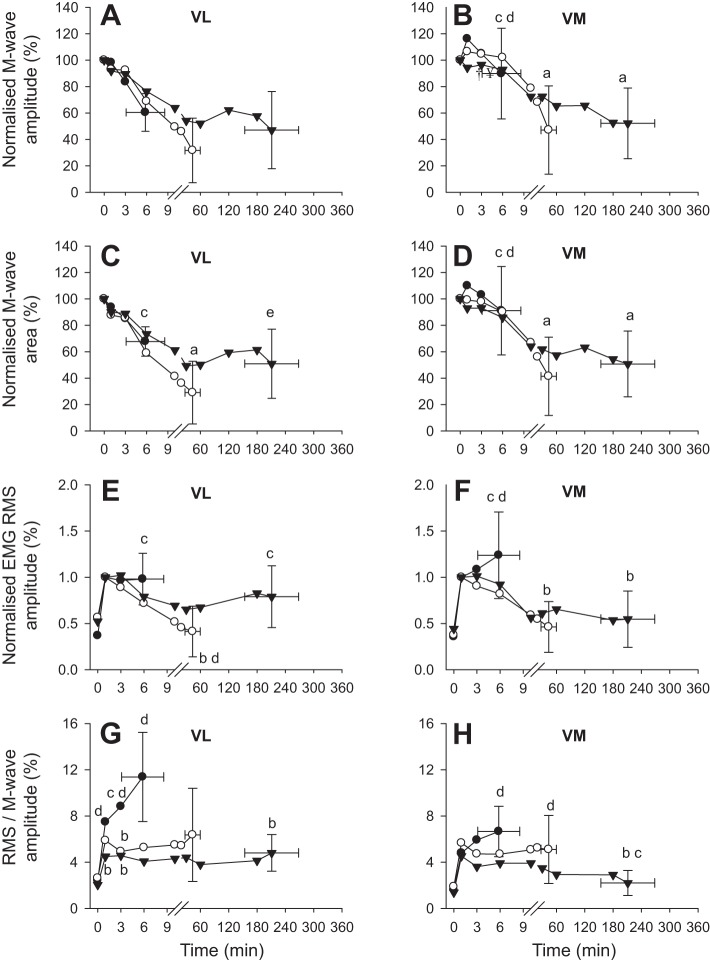
Normalized M-wave amplitude (*A* and *B*), M-wave area (*C* and *D*), voluntary EMG RMS amplitude (*E* and *F*), and RMS/M-wave amplitude (*G* and *H*) during severe- (solid circles), heavy- (open circles), and moderate-intensity (solid triangles) exercise in vastus lateralis (VL) and vastus medialis (VM) muscles. M-wave amplitude and area were normalized to maximum M-wave during baseline pedaling, EMG RMS was normalized to M-wave amplitude at 1 min of exercise, and RMS/M-wave was normalized to corresponding M-wave amplitude at each measurement time point. Error bars have been omitted from all but the final data point to aid clarity. ^a^Different from rest; ^b^different from severe-intensity (*P* < 0.05); ^c^different from heavy-intensity (*P* < 0.05); ^d^different from moderate-intensity (*P* < 0.05); and ^e^trend for difference from heavy-intensity (*P* = 0.055).

M-wave characteristics at T_lim_ for the three severe-intensity exercise tests, and for moderate-intensity, heavy-intensity, and the mean of the severe-intensity exercise tests are shown in Fig. 5, *A–D*. Peripheral neuromuscular excitability at T_lim_, indicated by M-wave amplitude and M-wave area, did not differ among the severe-intensity tests (all *P* < 0.05) (Fig. 5, *A* and *C*). M-wave amplitude and M-wave area at T_lim_ were greater during severe-intensity exercise compared with both heavy- and moderate-intensity exercise in the VM (*P* < 0.05), and the M-wave area at T_lim_ was also greater in severe- than in heavy-intensity exercise in the VL (*P* < 0.05) (Fig. 5, *B* and *D*). Differences in M-wave characteristics between severe-, heavy-, and moderate-intensity exercise at each measurement time point are shown in Fig. 6, *A–D*.

#### Voluntary activation and neural drive.

Voluntary muscle activation level, measured as EMG RMS amplitude, and neural drive, as indicated by RMS/M-wave amplitude, did not differ at T_lim_ among the severe-intensity exercise tests (all *P* < 0.05) (Fig. 5, *E* and *G*). Both EMG RMS and RMS/M were greater at T_lim_ for severe-intensity compared with heavy- and moderate-intensity exercise in VM (*P* < 0.05) (Fig. 5*F*). In VL, the EMG RMS at T_lim_ was also greater for severe- than for heavy-intensity exercise, and RMS/M was greater for severe- than for moderate-intensity exercise (both *P* < 0.05) (Fig. 5, *F* and *H*). The only difference in neuromuscular variables observed at T_lim_ between moderate- and heavy-intensity exercise was a significantly greater EMG RMS in VL (Fig. 5*F*). Differences in EMG RMS and RMS/M during severe-, heavy-, and moderate-intensity exercise at each measurement time point are shown in Fig. 6, *E–H*.

### Relationships Between Physiological and Neuromuscular Variables

During severe-intensity exercise, M-wave amplitude decreased in parallel with [PCr] depletion and plasma K^+^ accumulation (Table 2). Moreover, increased neural drive (RMS/M) was correlated with high blood [lactate] and plasma [K^+^] and low muscle [PCr], and high muscle [lactate] and [glycogen] (Table 2). During heavy-intensity exercise, the reduction in M-wave amplitude was related to low muscle [PCr] and high plasma [K^+^], and increased neural drive was related to high plasma [K^+^] and low muscle [PCr], and high muscle [lactate] and [glycogen] (Table 2). During moderate-intensity exercise, the M-wave amplitude was inversely correlated with the reduction in [PCr] (Table 2).

**Table 2.** Correlation coefficients between the rate of change in blood and muscle tissue variables and rate of change in neuromuscular variables measured in vastus lateralis muscle

	*M-Wave Amplitude*	*Voluntary EMG*	*Neural Drive*
Severe			
Blood [lactate]^b^	−0.30	0.57^a^	0.47^a^
Plasma [K^+^]^b^	−0.39^a^	0.68^a^	0.64^a^
[PCr]^c^	0.59^a^	−0.80^a^	−0.80^a^
[lactate]^c^	−0.40	0.44^a^	0.55^a^
[glycogen]^c^	−0.22	0.46^a^	0.56^a^
[pH]^c^	−0.13	0.36	0.37
[ATP]^c^	0.21	−0.60^a^	−0.59^a^
Heavy			
Blood [lactate]^d^	−0.42	0.13	0.49
Plasma [K^+^]^d^	−0.88^a^	−0.29	0.86^a^
[PCr]^e^	0.93^a^	−0.28	−0.72^a^
[lactate]^e^	−0.25	0.63	0.66
[glycogen]^e^	−0.15	0.53	0.77^a^
[pH]^e^	0.13	0.78^a^	0.27
[ATP]^e^	−0.26	0.32	0.63
Moderate			
Blood [lactate]^d^	0.08	0.05	0.10
Plasma [K^+^]^d^	0.12	0.18	0.49
[PCr]^e^	−0.67^a^	−0.36	0.58
[lactate]^e^	−0.44	−0.34	0.04
[glycogen]^e^	−0.10	0.43	0.23
[pH]^e^	0.19	0.06	−0.30
[ATP]^e^	0.09	0.59	0.24

a *P* < 0.05;

b *n* = 33;

c *n* = 24;

d *n* =10;

e *n* = 7.

## DISCUSSION

To our knowledge, the present study is the first to combine muscle biopsy, blood analyses, and measurements of neuromuscular excitability and neural drive (via electrical stimulation of the femoral nerve during exercise) to assess the muscle metabolic, acid-base, and neuromuscular responses to cycling performed within discrete exercise intensity domains (32). The data presented herein provide novel insight into the in vivo relationships between exercise intensity, muscle metabolic perturbation, and neuromuscular function, and support the notion that LT/GET and CP separate exercise intensity domains within which exercise tolerance is limited by discrete fatigue mechanisms. In classical terms, when exercise intensity exceeds CP, the oxidation of fat and carbohydrate cannot keep pace with required ATP turnover, and the rate of pyruvate production from glycolysis exceeds the capacity of the Krebs cycle, resulting in progressive increase in intramuscular lactate and H^+^ concentrations. We demonstrated that a similar muscle metabolic milieu (i.e., [ATP], [PCr], [lactate], and pH) was attained at T_lim_ irrespective of work rate within the severe-intensity domain. The muscle metabolic perturbation was greater (i.e., lower [ATP] and pH, and higher [lactate]) at T_lim_ following severe- and heavy-intensity exercise compared with moderate-intensity exercise. In contrast, more extensive muscle glycogen depletion occurred during moderate- compared with both severe- and heavy-intensity exercise.

However, although the results indicate that CP represents a critical threshold for both muscle metabolic control and neuromuscular fatigue development, the importance of GET in separating exercise intensity domains was less obvious; unlike some muscle metabolic, pulmonary gas exchange, and blood [lactate] responses, neuromuscular indices of fatigue development were not strikingly different between moderate-intensity and heavy-intensity exercise.

### Fatigue During Severe-Intensity Exercise

T_lim_ during the severe-intensity exercise tests ranged from 2.2 to 13.9 min, and in all cases, subjects achieved V̇o_2peak_. Historically, the amount of work that can be performed above CP (i.e., the curvature constant of the power-duration relationship, W′), and therefore the cause or causes of exercise intolerance within the severe-intensity domain, has been linked to the depletion of high-energy phosphates and a source related to anaerobic glycolysis, along with a finite amount of stored O_2_ (43, 44). Consistent with this, recent studies have demonstrated that, at least for small-muscle-mass exercise, the utilization of this finite energy store (W′) coincides with depletion of muscle PCr and accumulation of fatigue-related metabolites (i.e., P_i_, H^+^) until a consistent, presumably “limiting” value is attained (33, 69). The findings of the current study indicate that, irrespective of work rate or exercise duration (approximately 2–14 min), T_lim_ during severe-intensity exercise is associated with the attainment of consistently low values of muscle [PCr] (~23% of resting value), [ATP] (~76% of resting value), and pH (~6.56), and consistently high values of muscle [lactate] (~1,382% of resting value) and blood [lactate] (~838% of resting value). It should be noted that the observed muscle metabolite and substrate changes are reflective of the homogenate muscle sample, and therefore reflect the mean values for that muscle portion. It is known that the depletion of muscle [PCr] during exercise displays significant regional heterogeneity (13, 54). It is therefore possible that the eventual failure of subjects to maintain the requisite power output was caused by attainment of sufficiently low values of [PCr] and, perhaps, [ATP], and/or sufficiently high values of muscle metabolites ([P_i_], [ADP], [H^+^], and their sequelae) within some of the recruited muscle fibers [(51); see also (3, 23, 24)]. Clearly, subjects either could not, or would not, tolerate this “critical combination” of substrate and metabolite concentrations, but it is not possible to ascertain whether this was related to direct effects of the muscle metabolic milieu on contractile function (17) or to the attainment of some individual sensory “critical fatigue threshold” that might constrain central motor drive and muscle activation via feedback from type III/IV neural afferents (4). The appreciable metabolic perturbation we observed during severe-intensity exercise was associated with a concomitant decrease in M-wave amplitude in both VL and VM muscles. A strong inverse correlation was observed between both the voluntary EMG RMS amplitude and neural drive, and changes in [ATP] and [PCr] (Table 2). This is consistent with there being greater engagement of central neural mechanisms (e.g., muscle fiber recruitment and firing frequency modulation) to compensate for peripheral fatigue development.

We have proposed that changes in muscle metabolic status that occur concomitantly with expenditure of W′ drive the development of the V̇o_2_ slow component during severe-intensity exercise (8, 33, 70). Thus, exercise intolerance in this intensity domain is associated with the complete utilization of W′, attainment of some “critical” combination of muscle substrate and/or metabolite concentrations, and achievement of V̇o_2peak_ (8, 14, 47, 70). In the present study, we observed a reduction in muscle excitability in parallel with increased metabolic stress. The reduction in muscle membrane excitability is likely mediated, at least in part, by changes in plasma [K^+^] (Table 2), which may reflect a rise in interstitial [K^+^] within the t-tubule, which weakens propagation of the action potential along the surface membrane. Increased extracellular [K^+^] impairs force generation due to depolarization of the cell membrane, resulting in a reduced amplitude of the action potential (11, 40). This process attenuates Ca^2+^ release from the sarcoplasmic reticulum, reducing cross-bridge formation and the force-generating capacity of the myocyte (36). In our study, increased plasma [K^+^] was accompanied by a transient increase in neural drive that was brought about via a preservation of the EMG amplitude with reduced M-wave amplitude. It was notable that reductions in M-wave amplitude and M-wave area in the VM muscle during exhaustive severe exercise were less pronounced compared with moderate and heavy exercise (Fig. 5, *B* and *D*), suggesting that muscle excitability was preserved to a greater extent than at lower exercise intensities. It is important, however, to consider this finding in the context of increasing neural drive during severe exercise (Fig. 6, *G* and *H*), which implies that exercise cessation was not due to central fatigue. Low muscle pH attained during severe exercise may attenuate the reduction in muscle membrane excitability (3, 24). Furthermore, muscle glycogen content, a key regulator of sarcoplasmic Ca^2+^ release rate and thus muscle excitability (15, 50), did not fall significantly during severe exercise. Precisely how utilization of W′ and the associated alterations in muscle substrate and metabolite concentrations and ionic changes influence muscle excitability warrants further investigation.

### Fatigue During Heavy-Intensity Exercise

Heavy-intensity exercise was maintained for an average of 43.5 min (T_lim_ ranged from 20.5 to 67.4 min) and, in contrast to severe-intensity exercise, no subject achieved V̇o_2peak_ at T_lim_ (~87% V̇o_2peak_). Consistent with our second hypothesis, the muscle metabolic perturbation experienced following heavy-intensity exercise was less than that observed following severe-intensity exercise, but was greater than that observed following moderate-intensity exercise. At T_lim_, significant reductions were observed in muscle [PCr] (~66%), [ATP] (~12%), [pH] (~97%]), and [glycogen] (~59%), and there was a significant increase in muscle [lactate] (~447%) relative to resting values. Similarly, blood [lactate] and plasma [K^+^] displayed greater perturbation relative to moderate-intensity exercise, but less perturbation relative to severe-intensity exercise (Fig. 3). It is of interest that the decrease in muscle excitability from rest to T_lim_ was greater during heavy-intensity than during severe-intensity exercise (Fig. 5). Following the onset of exercise, plasma [K^+^] increased rapidly to attain a peak value at 10 min, which was sustained until T_lim_; the reduction in M-wave amplitude followed a similar temporal profile. It is therefore likely that the initial reduction in M-wave amplitude was a result of plasma [K^+^] accumulation, which reduced the release of Ca^2+^ from the sarcoplasmic reticulum, thus impairing excitation-contraction coupling (36, 71). As heavy-intensity exercise continued, it is possible that the combined metabolic and ionic perturbation, coupled with the ~60% decrease in muscle [glycogen], may have further impaired Ca^2+^ release and cross-bridge formation (2, 3, 23, 24, 36, 40, 41) and/or the sensitivity of the myofilaments to Ca^2+^ (17). Although fatigue development during heavy-intensity exercise appears to be more complicated than it is for severe-intensity exercise, it is related to the combined influence of ionic changes in muscle membrane excitability, muscle metabolite accumulation, and the decrease in energy substrate, which act collectively to impair excitation-contraction coupling.

### Fatigue During Moderate-Intensity Exercise

Moderate-intensity exercise performed at a work-rate of 20 W below GET was continued for an average of 211 min, with subjects working at ~52% V̇o_2peak_ at T_lim_. Muscle metabolic perturbation was relatively slight in this domain (Fig. 3). For example, at the end of exercise, muscle [PCr] had fallen to ~76% of the baseline value, and pH had fallen by 0.1 unit from the resting value, and blood [lactate] and plasma [K^+^] were also largely unchanged (Figs. 3 and 4). There was, however, a large reduction (–83%) in muscle [glycogen] (1, 29, 59, 60). It is therefore likely that the development of peripheral fatigue within the moderate-intensity domain is related to depletion of muscle glycogen and impairment in neuromuscular excitability and transmission (15, 28, 49, 50, 62). In addition to being an essential substrate for regeneration of ATP, it has been demonstrated that under conditions in which [ATP] is held high, low muscle [glycogen] can impair muscle function (49, 62). The association between low muscle [glycogen] and impaired muscle function can be attributed to the modulatory role of glycogen in the release of Ca^2+^ from the sarcoplasmic reticulum (15, 19, 20, 28, 49, 50). In keeping with the role of glycogen in excitation-contraction coupling, individuals with glycogen phosphorylase deficiency (McArdle disease) do not experience a considerable drop in pH; rather, they demonstrate an earlier decline in the M-wave amplitude during exercise (16). Furthermore, glucose administration during exercise has been shown to partially restore both the M-wave amplitude and muscle contractility (34, 37, 63), thus supporting the notion that carbohydrate availability modulates muscle excitability and contractile function. The findings of the present study show that moderate-intensity exercise (<GET) can be sustained for a long duration with little change in muscle metabolites and indicate that muscle glycogen depletion is the likely mechanism responsible for the decline in neuromuscular function and exercise intolerance in this domain.

Most research investigations of neuromuscular fatigue development during exercise have focused on small muscle groups and have been limited to the assessment of neuromuscular function before exercise and as soon as possible (usually within 2–3 min) after exercise. Considering the task-specific nature of neuromuscular fatigue development and the rapid recovery in muscle function (within 2 min) after high-intensity cycle exercise (26), it is possible that previously reported changes in neuromuscular function from before exercise to after exercise underestimate fatigue development during exercise. Recently, Sidhu et al. (56) adopted an approach that uses the motor compound action potential (M-wave) for assessment of changes in neuromuscular function during cycle exercise. By adopting a similar approach (56), we found large reductions in M-wave amplitude and M-wave area in both VL and VM muscles during exercise to T_lim_ in each discrete exercise intensity domain. This suggests that changes in muscle excitability linked to the fatigue process can occur consequent to a wide range of perturbations in muscle and blood chemistry, with limited differentiation between exercise intensity domains. The consistency of indices of neuromuscular fatigue during severe-intensity cycling exercise in our study contrasts with a recent report by Thomas et al. (65) in which peripheral fatigue, assessed after exercise using electrical stimulation during isometric contractions, was greater at higher work rates within the severe-intensity domain. It is possible that this reflects differences in the experimental techniques employed, and underlines the importance of accounting for the task specificity of fatigue and the dynamics of muscle recovery after exercise (10).

### Conclusion

This study employed a novel and rather comprehensive combination of invasive and noninvasive techniques that enabled simultaneous assessment of metabolic, ionic, systemic, and neuromuscular factors that define muscular performance. Although direct measures of the contribution of central factors to fatigue were not employed, peripheral nerve stimulation permitted elucidation of their relative importance in neuromuscular fatigue development during exhaustive cycle exercise performed within each of the well-defined exercise intensity domains. This study is consistent with the notion that GET and CP demarcate exercise intensity domains within which fatigue is mediated by distinct mechanisms. Exercise intolerance within the severe-intensity domain (>CP) was associated with the attainment of a consistent critical muscle metabolic milieu (i.e., low [PCr] and pH). In contrast, moderate-intensity exercise (<GET) was associated with more significant depletion of muscle [glycogen]. The causes of fatigue during heavy-intensity exercise (>GET, <CP) were more obscure with intermediate changes in muscle metabolic perturbation and glycogen depletion being apparent. These results are consistent with the notion that both GET and CP demarcate exercise intensity domains characterized by distinct respiratory and metabolic profiles. Strikingly, CP represents a boundary above which both metabolic and neuromuscular responses conform to a consistent ceiling or nadir irrespective of work rate and exercise duration.

## DISCLOSURES

No conflicts of interest, financial or otherwise, are declared by the authors.

## AUTHOR CONTRIBUTIONS

M.I.B., A.M.J., S.J.B., K.J.M., J.L.B., and A.V. conceived and designed research; M.I.B., A.M.J., J.R.B., S.J.B., L.J.W., S.T.J.M., C.T., J.K., and A.V. performed experiments; M.I.B., J.R.B., L.J.W., C.T., J.K., K.J.M., and J.L.B. analyzed data; M.I.B., A.M.J., J.R.B., S.J.B., P.S., K.J.M., J.L.B., and A.V. interpreted results of experiments; M.I.B. and A.V. prepared figures; M.I.B. and K.J.M. drafted manuscript; M.I.B., A.M.J., L.J.W., K.J.M., J.L.B., and A.V. edited and revised manuscript; M.I.B., A.M.J., J.R.B., S.J.B., L.J.W., S.T.J.M., C.T., J.K., P.S., K.J.M., J.L.B., and A.V. approved final version of manuscript.
